# DNA-dependent RNA polymerase incorporates β-D-N4-hydroxycytidine (NHC) linking Molnupiravir to host transcription-dependent mutagenesis

**DOI:** 10.1016/j.jbc.2026.111409

**Published:** 2026-03-26

**Authors:** Nathalie Garnier, Hongtao Xu, Raymond F. Schinazi, Baek Kim

**Affiliations:** Center for ViroScience and Cure, Department of Pediatrics, Emory University School of Medicine and Children’s Healthcare of Atlanta, Atlanta, Georgia, USA

**Keywords:** DNA-dependent RNA polymerase, HIV-1, mutation synthesis, NHC-TP, reverse transcriptase

## Abstract

Molnupiravir, a prodrug of β-D-N^4^-hydroxycytidine (NHC), is an antiviral RNA mutagen that is incorporated by viral RNA-dependent RNA polymerases (RdRp) during replication of viral RNA genomes, ultimately driving target viruses such as SARS-CoV-2 toward lethal mutagenesis. In this study, first, we biochemically tested whether DNA-dependent RNA polymerases (DdRps) including T7 RNA polymerase and host RNA polymerase II, can also incorporate NHC-triphosphate (NHC-TP) during RNA synthesis from double-stranded DNA (dsDNA) templates. *In vitro* transcription (IVT) by T7 RNA polymerase was evaluated under two conditions: (1) all four natural ribonucleoside triphosphates (rNTPs) and (2) three natural rNTPs (ATP, GTP, and UTP) supplemented with NHC-TP. Full-length RNA products were generated in both conditions, indicating that T7 RNA polymerase incorporates NHC-TP during DNA-dependent RNA synthesis. Second, these IVT-derived RNA products were subsequently reverse-transcribed into single-stranded DNA (ssDNA) using HIV-1 reverse transcriptase (RT). Comparable ssDNA yields were also obtained from both RNA templates, suggesting that NHC-monophosphates embedded in RNA template do not affect the RNA-dependent DNA polymerase (RdDp) activity of HIV-1 RT under the experimental conditions tested. Third, next-generation sequencing (NGS) analysis of the reverse-transcribed products revealed the expected NHC-mediated C to T transition mutations, confirming the mutagenic impact of NHC during the HIV-1 RT-mediated RdDp reactions. Finally, incorporation of NHC-TP by human RNA polymerase II was further confirmed using IVT reactions performed with HeLa cell nuclear extracts. Overall, these biochemical investigations establish both the capacity of DdRps to incorporate NHC-TP and the characteristic mutagenic signature induced by NHC during HIV-1 RT-mediated RNA-dependent DNA synthesis.

Molnupiravir, a prodrug of β-D-N^4^-hydroxycytidine (NHC), is a ribonucleoside analog antiviral (1, 2, 3), and NHC is an RNA mutagen that induces lethal mutagenesis to various RNA viruses including SARS-CoV-2. Upon the transport of NHC to the cells, NHC is converted to NHC-triphosphate (NHC-TP), its active drug form, by cellular nucleoside/nucleotide kinases. NHC-TP is incorporated into nascent viral RNAs by viral RNA-dependent RNA polymerases (RdRp) during viral replication (1). Two different tautomers of NHC base induce mis-base-pairing and then replicative errors: the hydroxylamine form as a CTP mimic base-pairs with guanine, and the oxime form as an UTP mimic base-pairs with adenosine (4, 5). The hydroxylamine form has been reported the more predominant form (6). Subsequently, during the replication of the NHC-monophosphate (NHC-MP) containing viral RNAs, viral RdRps frequently recognize NHC-MPs in viral RNA templates as U instead of C, leading to the incorporation of ATP instead of GTP to the newly synthesized RNA products. When these newly synthesized RNAs are replicated, the C to U transition mutations is generated at the C sites where NHC-MPs were incorporated. This C to U mutagenesis can repeatedly occur through the RNA genome replications, accumulating many of this transition mutation. The uncontrolled mutation accumulation in viral RNA genomes can lead to lethal mutagenesis of the viral populations, also called error catastrophe, rendering replication-incompetent viral populations (4, 5, 7, 8, 9, 10, 10, 10, 11, 12). It has also been demonstrated that, once incorporated into the coronavirus RNAs, NHC-MPs in the RNA template are less susceptible to drug-resistant mutations and proofreading removal by viral exonuclease (13), showing comparable activity against wild-type and exonuclease-deficient viruses. This may explain why NHC-TP can surpass the error threshold without being repaired, resulting in a mutation rate that exceeds the level required to maintain viral viability.

RNA mutagens, like NHC, have been investigated mainly for targeting RdRps of RNA viruses. However, their impacts on cellular RNA polymerases remain to be fully understood. Cells harbor various RNA polymerases consuming rNTPs for producing various types of cellular RNAs such as mRNAs, tRNAs, and ribosomal RNAs (14). However, all cellular RNA polymerases are DNA-dependent RNA polymerases (DdRp). In fact, NHC-TP can be incorporated to RNAs by the human mitochondrial DdRp (PolRMT) without causing major cell cytotoxicity (15) even though whether other major cellular RNA polymerases such as RNA polymerase II can incorporate NHC-TP remains to be investigated.

Unlike other RNA viruses, retroviruses including HIV-1 use the host-cell RNA polymerase II for their RNA genome synthesis (16). Therefore, it is plausible that if host RNA polymerase II incorporates NHC-TP during HIV-1 transcription in the infected cells, these NHC-MPs incorporated in HIV-1 RNA genomes can also generate mutations in HIV-1 proviral DNAs during reverse transcription process at the newly infected cells. HIV-1 reverse transcriptase (RT), an RNA-dependent DNA polymerases, can mis-incorporate dATP instead of the canonical dGTP at the NHC-MP sites of viral RNA genomes during the (−) strand DNA synthesis of reverse transcription. However, whether RTs recognize NHC-MPs incorporated in viral RNA templates as a mutagen during DNA synthesis remains untested.

In this study, we conducted a comprehensive biochemical simulation study to determine whether DdRps such as T7 RNA polymerase and the host RNA polymerase II also incorporate NHC-TP during *in vitro* transcription (IVT) from dsDNA templates. We further investigated whether HIV-1 RT recognizes NHC monophosphates (NHC-MPs) embedded within RNA templates as mutagenic nucleotides during its RNA-dependent DNA polymerization (RdDp). Overall, these simulation analyses delineate the mechanistic basis underlying both the incorporation capacity of DdRps for NHC-TP and the mutagenic consequences of NHC during RT-mediated RdDp reactions.

## Results

### Incorporation of NHC-TP by T7 RNA polymerase

**Figure 1:**
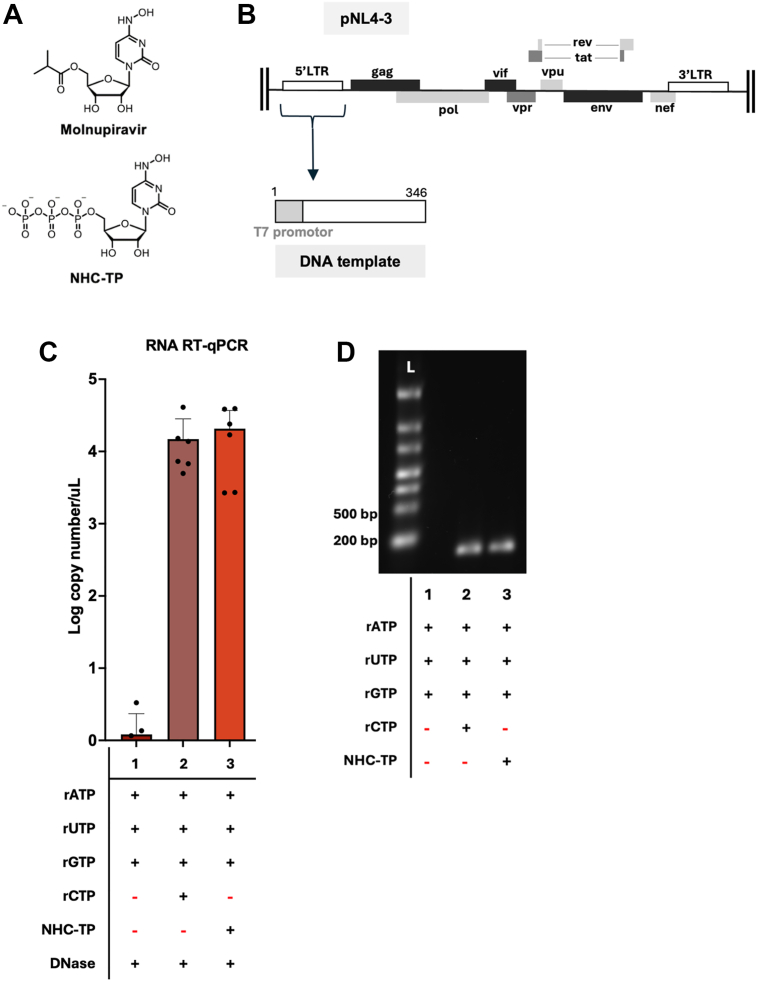
**Incorporation of NHC-TP during T7 RNA polymerase-mediated *in vitro* transcription (IVT)**. (*A*) Chemical structures of Molnupiravir and NHC-triphosphate. (*B*) Scheme of the 346 bp DNA template encoding T7 promoter sequence and a 326 bp sequence from the 5′ LTR region of HIV-1 pNL4-3 plasmid for T7 polymerase-mediated IVT reactions. (*C*) RNA copy numbers after qRT-qPCR performed with extracted RNA products from IVT reactions after DNase treatment. The T7 polymerase-mediated IVT reactions were conducted in triplicates, and each experimental replicate is analyzed by qRT-PCR in two technical replicates. Mean with SD are represented with bars. (*D*) Agarose gel electrophoresis of one representative IVT products after DNase treatment. L: RNA ladder. IVT reactions were conducted with three different nucleotide pool compositions: (1) 3 rNTPs (ATP, GTP, and UTP) without CTP (negative control), (2) four rNTPs (ATP, GTP, UTP, and CTP: positive control), and (3) the 3rNTP (ATP, GTP and UTP) and NHC-TP. The nucleotide concentrations used were their cellular concentrations previously reported (17, 18).

First, we tested whether T7 RNA polymerase, a DdRp, can incorporate NHC-TP, the active form of Molnupiravir (Fig. 1*A*) during IVT reactions. For this test, we generated a DNA template encoding a 328 nt region at the 5′ LTR of pNL4-3, a molecular clone of HIV-1 NL4-3 strain, fused to T7 promoter (Fig. 1*B*). Next, we conducted T7 RNA polymerase mediated IVT reactions with three different kinds of rNTP compositions (Fig. 1*C*): 1) only 3 rNTP without CTP (negative control), 2) all four natural rNTPs at their concentrations found in cells (17) (positive control) and 3) three natural rNTPs (ATP, GTP, and UTP) with replacement of CTP with NHC-TP at its concentration that we found in cells treated with NHC nucleoside (18). Note that the IVT reaction with three natural rNTPs with NHC-TP forces T7 RNA polymerase to incorporate NHC-TP at all CTP incorporation sites (C sites) in the DNA template during RNA synthesis. Next, we compared the copy numbers of the *in vitro* transcribed RNA products by qRT-PCR after the extraction of the RNA products from the IVT reactions (Fig. 1*C*). However, since the DNA template used for the IVT reaction was also extracted together with the RNA products, to accurately measure the copy numbers of the RNA products in the extracted RNA products, we removed the DNA template included in the extracted RNAs by DNase treatment, which reduced the DNA template copy numbers by ∼10^4^ folds (Fig. S2) as measured by qPCR before and after DNase treatment. Indeed, as shown in Figure 1*C*, the IVT reaction with the three natural rNTPs with the replacement of CTP with NHC-TP produced high RNA product copy numbers, compared to the negative control reaction (with only 3 rNTPs), and its copy number were comparable with those of the IVT reaction with four natural rNTPs (positive control). Next, we analyzed the RNA products in agarose gel electrophoresis (Fig. 1*D*), and we also visually detected a similar amount of the expected RNA products between the IVT reactions with all four rNTPs and the ones with three rNTPs and NHC-TP. Therefore, these data support that T7 RNA polymerase incorporates NHC-TP during its DNA-dependent RNA polymerization IVT reactions under our *in vitro* condition.

### cDNA synthesis by HIV-1 RT from NHC-monophosphate containing RNA template

**Figure 2:**
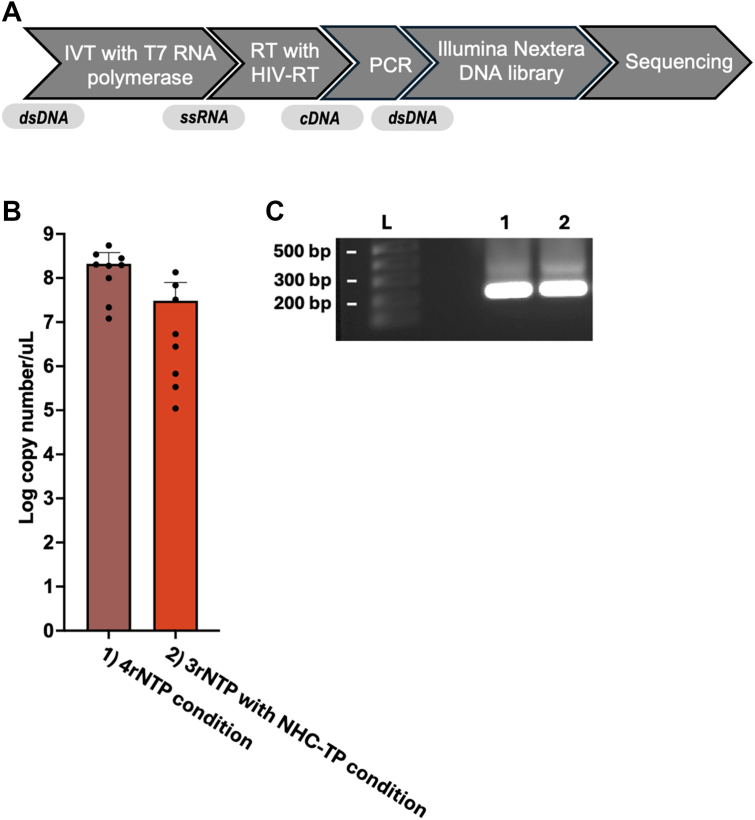
**cDNA synthesis from NHC-MP-containing RNA template by HIV-1 reverse transcriptase (RT)**. (*A*) Scheme to summarize the experimental flow to investigate the NHC-mediated mutagenesis in this study. (*B*) cDNA levels in the HIV-1 RT reactions with RNA products synthesized by T7 RNA polymerase at two nucleotide pool conditions, (1) four rNTPs (ATP, GTP, UTP, and CTP) and (2) 3 rNTPs (ATP, GTP, and UTP) with NHC-TP. IVT products in triplicates were reverse transcribed by HIV-1 RT, and the RT products were analyzed by qPCR in duplicates. Mean with SD are represented with bars. (*C*) Agarose gel electrophoresis of one representative amplified DNA product. L: DNA ladder.

Next, since we confirmed that T7 RNA polymerase incorporates NHC-TP during its IVT reaction, we tested whether HIV-1 RT can generate mutations during DNA synthesis from the NHC-monophosphate (NHC-MP)-containing RNA template produced by T7 RNA polymerase. The overall workflow of this test was summarized in Figure 2*A*.

First, we investigated whether NHC-MPs embedded in RNA template affect the DNA synthesis by HIV-1 RT. For this test, we employed the 328 nt RNA products from the IVT reactions with the two rNTP compositions (all four rNTPs vs. 3 rNTPs with NHC-TP) earlier described in Figure 1. We performed RT reactions with HIV-1 RT protein purified in our laboratory (19) and measured cDNA products by qPCR and agarose gel analysis. As shown in Figure 2*B*, HIV-1 RT produced similar cDNA copy numbers from the NHC-MP containing RNA template with those from the IVTs with four natural rNTPs, which was also confirmed with the agarose gel analysis (Fig. 2*C*). This data suggests that NHC-MPs embedded in RNA templates do not significantly affect the processive DNA synthesis during RdDp reactions by HIV-1 RT under our RT reaction condition.

### Mutation synthesis during RdDp reactions of HIV-1 RT from NHC-MP-containing RNA template

Next, we investigated the mutation synthesis that could occur during the cDNA synthesis by HIV-1 RT from the NHC-MP containing RNA templates by using next-generation sequencing (NGS) method. For this test, an equal amount of IVT products from the two different NTP pool compositions; 1) all four NTPs and 2) 3 NTPs (ATP, GTP and UTP) with NHC-TP, was amplified to generate the dsDNA libraries, which were sequenced on the MiSeq instrument (Fig. 2*A*). The 210 bp region of the DNA template described in Figure 1*B* was amplified by PCR, and this 210 bp PCR product was used as a reference control to monitor the background mutation rate during the NGS process (Fig. S1). The raw sequencing data were applied to the Galaxy server to filter Illumina error: removing adapter with the trimming and mapping DNA sequences generated the reference sequence, which is identical with the original DNA template sequence. In our sequencing data analysis, we employed Ivar variant method to detect mutations: Ivar variant method mainly detects frequent and abundant mutations in consensus sequences, present at higher frequencies (>5%) (20), which is the case with the RNA template synthesized with 3 rNTPs (ATP, UTP and GTP) and NHC-TP.

**Figure 3:**
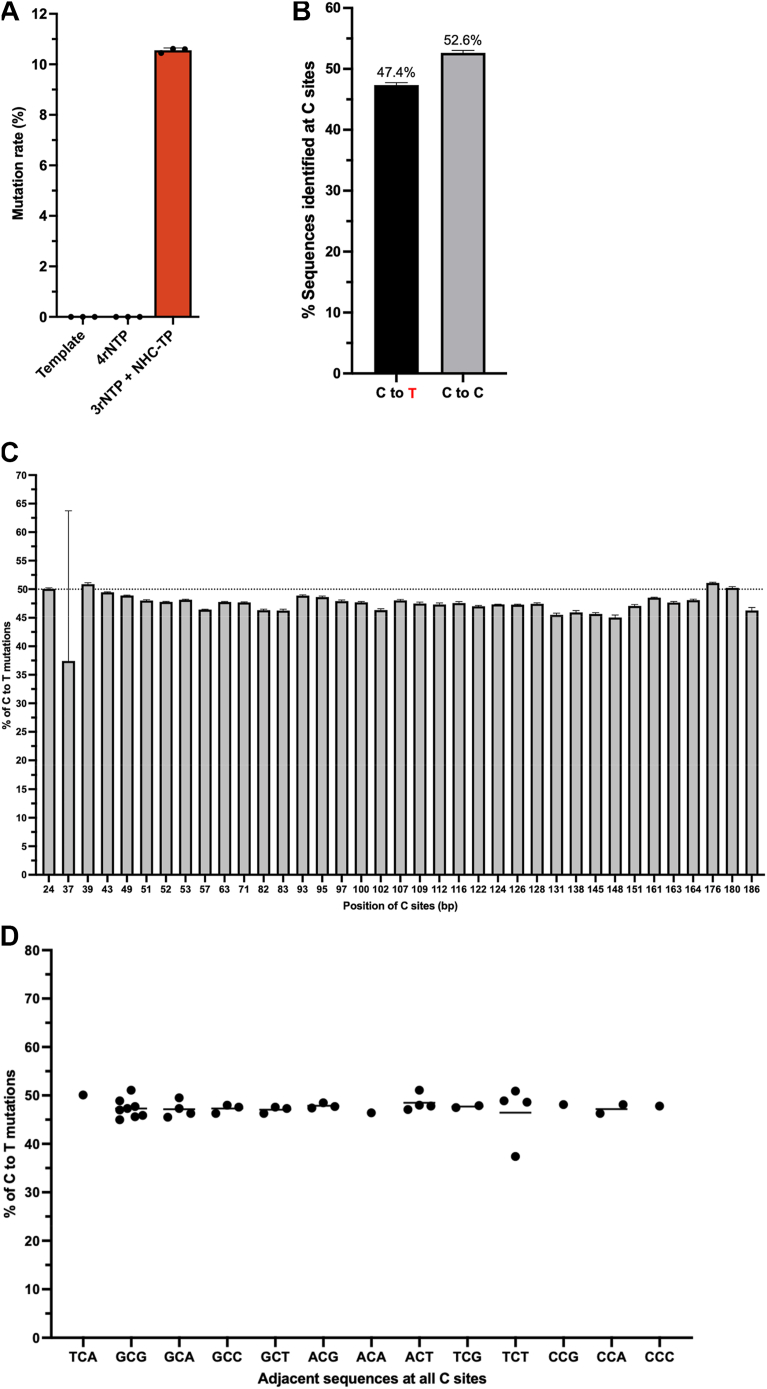
**Mutation rate and spectrum observed in reverse transcription products of RNA template generated by T7 RNA polymerase-mediated IVT**. (*A*) Mutation rates determined by NGS with IVT products generated from Figure 2, *B* and *C* at the two nucleotide pool conditions (4 rNTPs and 3 rNTPs with NHC-TP) and the DNA template used for the IVT. The sequencing data were analyzed by Ivar method, and ratios between total mutation reads and total sequence reads were used to calculate mutation rates. The IVT RNA products generated in triplicates and DNA template were analyzed for sequencing. (*B*) Percentage of C to T mutations identified at C sites. These values were calculated by ratios between mutated reads at C sites and total reads at C sites. (*C*) Percentage of mutations at all C cites in the analyzed template. Numbers indicate the positions of Cs in this template sequence analyzed. (*D*) Percentage of mutations at all C cites with different adjacent nucleotide sequences. All data details are presented in Table S3.

First, the direct sequencing of the control dsDNA template provides the background mutation rate of our sequencing protocol. Indeed, as expected, no mutation was detected in the sequencing data with the reference DNA template when the data was analyzed by Ivar variant method (Fig. 3*A*, Table S3*A*), supporting the low background sequencing errors analyzed by this method that focuses on only frequent and abundant mutations. Next, the sequencing data with the library made with the IVT RNA product with all four natural rNTPs provides the mutation rate during the entire experimental process including RNA synthesis by T7 RNA polymerase (IVT), cDNA synthesis by HIV-1 RT, and PCR library construction as well as sequencing errors. Note that HIV-1 RT is an error-prone RT with an *in vitro* with 10^-4^∼10^-5^ mutation rates (21). When the data analyzed by Ivar variant method, the library generated from the RNAs produced with all four natural rNTPs gave 5.53 x 10^-6^ mutation rate (738 mutations among 417,075,142 nucleotides sequence, Fig. 3*A* and Table S3*A*). This is also a relatively low mutation rate likely because the Ivar method counts mainly frequent mutations. Next, we analyzed the sequencing data from the RNAs generated with three natural rNTP with NHC-TP (Fig. 3*A* and Table S3*A*) using the Ivar variant methods. Note that all C sites in this RNA template are replaced with NHC-MPs, and therefore all C cites in this RNA template are mutable. First, the calculated mutation rate was 1.1 x 10^-1^, which is ∼5 x 10^5^ times higher, compared to the mutation rate of the all four natural rNTP condition: this extremely high mutation frequency was expected because NHC-TP was forced to be incorporated at all C sites during RNA synthesis by T7 RNA polymerase.

Next, the mutation spectrum at the C sites was analyzed with the sequencing data from the IVT with 3 rNTPs and NHC-TP. As shown in Figure 3*B*, we observed the 52.6% canonical NHC-mediated C to T transitions and 47.4% no mutations (C to C) at the C sites (Fig. 3*B*). This observation supports that NHC-MPs in RNA template also induce the C to T transition mutagenesis during RdDp (RT) by HIV-1 RT, and the mutation potential of NHC-MPs embedded in this RNA template is 47.4% during the reverse transcription by HIV-1 RT.

Next, we analyzed the mutation frequency among the C (NHC) sites in the template synthesized with 3 rNTPs and NHC-TP (Table S3*B*). As shown in Figure 3*C*, no significant frequency variations among all C sites of the template, confirming the absence of mutational hot and cold spots among the C sites during the RT reaction. This finding supports that the nucleotide sequences adjacent to the NHC sites do not significantly affect the NHC-MP-mediated mutation synthesis when they are reverse-transcribed by HIV-1 RT, which is shown in Figure 3*D*. In fact, the adjacent nucleotide sequences at the NHC-TP incorporation sites of SARS-CoV2 RNAs affect the NHC-mediated mutation potential when the viral RNAs isolated from Molnupiravir treated patients (22). Possibly, while the adjacent nucleotide sequences can affect the NHC-TP incorporation efficiency during RNA synthesis, it is possible that the mutation synthesis is not affected by the adjacent sequence during the nucleotide incorporation opposite to the NHC-MPs already incorporated in RNA templates.

### Incorporation of NHC-TP by cellular RNA polymerase II using HeLa nuclear extract

Finally, we biochemically tested whether cellular RNA polymerase II also incorporated NHC-TP during RNA synthesis. For this test, we employed IVT reactions with HeLa cell nuclear extract (NE) that has been used for the Pol II mediated RNA synthesis (23). In this test, we constructed a DNA template encoding the ∼2 kb long 5′ LTR sequence of pNL4-3 encoding the HIV-1 promoter sequence that is transcribed by cellular RNA polymerase II in infected cells (24, 25).

**Figure 4:**
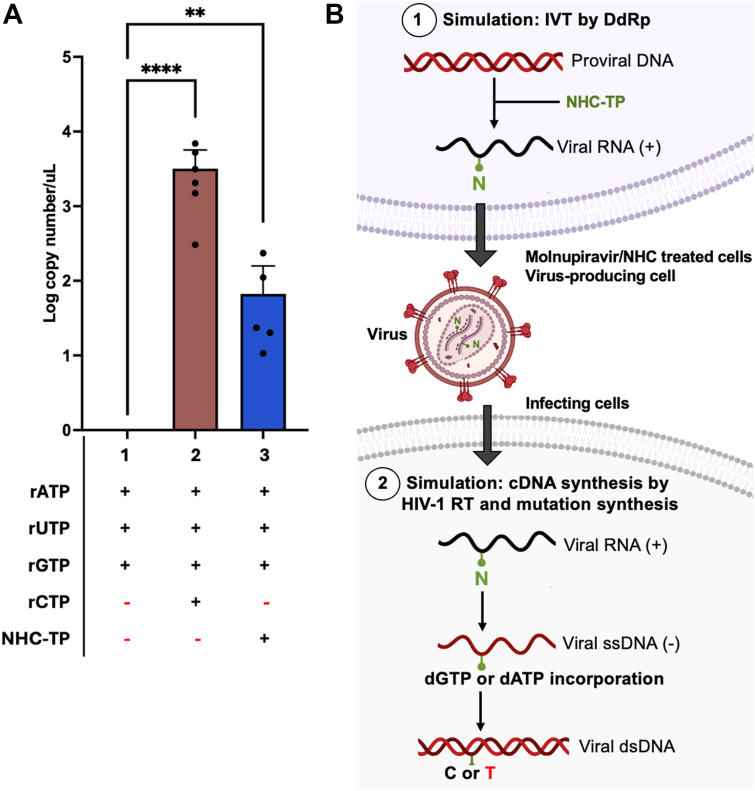
**Incorporation of NHC-TP by human RNA polymerase II in HeLa cell nuclear extract and model for RNA mutagen impact on HIV-1 replication**. (*A*) RNA copy numbers in IVT products with HeLa cell nuclear extract and three nucleotide pool compositions; (1) only 3 rNTPs (negative control), (2) all four rNTPs (positive control), and (3) 3 rNTPs with NHC-TP. The IVT products were treated with DNases before qRT-PCR analysis. The DNA template used in this IVT encodes the 5′LTR sequence of HIV-1 NL4-3 proviral DNA containing HIV-1 promoter transcribed by host RNA polymerase II. The nucleotide concentrations used were their cellular concentrations previously reported (17, 18). The IVT reactions were conducted in triplicates, and qRT-PCR analysis were conducted in duplicates per IVT reaction. Mean with SD are represented with bars. Statistical test non-parametric with Kruskal-Wallis test followed by Dunn’s test with *p*-value > 0.1234, ∗∗: *p*-value > 0.0021, ∗∗∗∗: *p*-value < 0.0001. Kruskal-Wallis test was significative with a *p*-value of 0.0002. Dunn’ test for comparing composition 1 and 2 was significative with *p*-value of <0.0001, and for comparing composition 1 and 3 was significative with *p*-value of 0.0078. (*B*) Model for RNA mutagen impact during HIV-1 replication. First, when the HIV-1 infected cells are treated with a RNA mutagen such as Molnupiravir, host RNA polymerase II incorporates NHC-TPs during HIV-1 transcription from the integrated proviral DNAs, releasing viral particles harboring viral RNA genomes containing NHC-MPs. This process is simulated in the experiments described in Figures 1 and 4*A* (Simulation by IVT by DdRps). Next, when these produced viral particles infect new cells, HIV-1 RT can incorporate dATP at C sites instead of dGTP during the first (−) strand DNA synthesis, generating C to T mutation in the (+) strand of the proviral DNAs before integration, which was simulated in the experiments described in Figures 2 and 3 (Simulation by cDNA synthesis by HIV-1 RT and mutation synthesis). This process can repeatedly occur during multiple infection cycles, which accumulates many mutations in viral genomes, leading to lethal mutagenesis. N corresponds to NHC-MP incorporation sites.

First, we performed the HeLa cell NE mediated IVT reactions with the same three rNTP compositions used for the T7 polymerase-based IVT assay (Fig. 1). Second, we treated RNA products with DNase to remove the DNA template (Fig. S3), and the RNA products were measured by qRT-PCR. As shown in Figure 4*A*, the RNA production with all four natural rNTPs (positive control) was much higher than the product with only three rNTP (minus CTP, negative control), indicating that RNA polymerase II in the HeLa cell NE was able to synthesize the target RNA from the HIV-1 promoter in the DNA template. Importantly, we also observed significant RNA products with three natural rNTPs (ATP, GTP, and UTP) with NHC-TP, compared with the negative control even though the RNA level from the three rNTPs with NHC-TP was lower than the RNAs from the positive control. Overall, the data from the HeLa cell NE mediated IVT reactions supports a possibility that cellular RNA polymerase II can also incorporate NHC-TP during transcription in the Molnupiravir-treated cells.

## Discussion

RNA mutagens have been developed as antiviral agents capable of inducing viral lethal mutagenesis, specifically against RNA viruses containing RNA-dependent RNA polymerases (26, 27, 28). Molnupiravir, a prodrug form of NHC, has been extensively investigated for its antiviral activity against various RNA viruses, including SARS-CoV-2 (29, 30), Influenza virus (30, 31), HCV (32), Chikungunya virus (18, 33), Venezuelan equine encephalitis virus (34), Jamestown Canyon virus and Cache Valley virus (35). These viral RNA-dependent RNA polymerases incorporate NHC-TP during replication, leading to the accumulation of the C to U transition mutations and ultimately to lethal mutagenesis. The effectiveness of molnupiravir has also been evaluated in clinical trials for COVID19 treatment (36, 37, 38, 39, 40, 41, 42, 43, 44, 45, 46, 47).

In this biochemical simulation study, first, we demonstrated that DNA-dependent T7 RNA polymerase is also capable of incorporating NHC-TP during RNA synthesis from DNA templates. Second, we showed that NHC-MPs embedded in RNA templates can also induce C to T transition mutations in DNA when these RNA templates are reverse-transcribed by HIV-1 RT (RdDp). Third, HIV-1 RT almost equally incorporates mutation-inducing dATP and non-mutation inducing dGTP opposite to the NHC-MP sites embedded in RNA template, resulting in the 50% probability of the C to T mutation synthesis at the NHC-MPs sites. Fourth, HIV-1 RT does not exhibit noticeable sequence specificity in mutation synthesis at nucleotides adjacent to NHC-MPs in the RNA template. Finally, this study shows that NHC-TP can be also incorporated by cellular DNA-dependent RNA polymerase II present in HeLa cell NE during transcription from the HIV-1 promotor.

Several studies have shown that NHC-TP is incorporated much less efficiently than CTP (1, 6, 48). In particular, a previous primer-extension-based nucleotide incorporation kinetic study with SARS-CoV-2 RNA polymerase complex (6) reported that NHC-TP is 30 times less efficiently incorporated than CTP. Primer extension–based assay is not technically feasible for DdRps because these polymerases initiate RNA synthesis in a primer-independent manner from their promoter sequences (49, 50). Therefore, we performed comparative T7 RNA polymerase-based IVT reactions using varying concentrations of either CTP or NHC-TP while keeping the concentrations of the other three nucleotides constant (Fig. S4). We observed that IVT reactions containing CTP still generated more product even after a 1/50 dilution compared with reactions containing NHC-TP. This result indicates that T7 RNA polymerase also incorporates NHC-TP at least 50-fold less efficiently than CTP, consistent with observations reported for SARS-CoV-2 RdRp. In addition, since HeLa nuclear extracts have been extensively used for the sources of RNA polymerase II complex for the *in vitro* study (49, 50, 51, 52), and we also employed the HeLa cell nuclear extract for our study, rather than undertaking the technically challenged task of purifying the human RNA polymerase complex that comprises of at least 12 subunits and requires multiple separate transcription factors for accurate simulation of HIV-1 LTR driven transcription (53). Indeed, the IVT reactions with HeLa cell nuclear extract (Fig. 4*A*) indicate that the reaction with three rNTPs and NHC-TP produced lower RNA levels than the reaction with all four rNTPs. This lower RNA synthesis could result from the lower incorporation efficiency of NHC-TP by RNA polymerase II, compared to CTP.

Next, the same biochemical study (6) also reported that SARS-CoV-2 RNA polymerase complex equally incorporates GTP and ATP at the NHC-MP sites in RNA template during RNA synthesis, and we also observed the equal dGTP and dATP incorporation at the NHC-MP sites of the RNA template by HIV-1 RT during DNA synthesis, as identified in the sequencing data (Fig. 3*B*).

Any RNA nucleotide mutagens that are incorporated into natural CTP with equal efficiency could induce significant toxicity by disrupting the intrinsic functions and structures of cellular RNAs produced in the treated cells. In fact, NHC-TP is incorporated less efficiently than other RNA-dependent RNA polymerases (1, 6, 54), thereby minimizing its potential toxicity. Due to the duality of its base-pairing, NHC-MPs in mRNAs can affect codon base pairing, leading to the incorporation of incorrect amino acids during translation. NHC-MPs could be detrimental to small cellular RNAs that function as their unique structures engineered by highly complex base-base pairing mechanisms such as tRNAs. Furthermore, since cells synthesize dNDPs from rNDP *via* ribonucleotide reductase (RNR) during *de novo* dNTP synthesis (55), the potential conversion of NHC-TP to dNHC-TP by RNR, which could induce unwanted cellular genotoxicity and DNA mutations, is another concern (4, 48, 56, 57).

Importantly, our biochemical simulation study employing NHC-TP proposes that RNA nucleoside/nucleotide mutagens could serve as a platform for potential anti-HIV-1 agents capable of inducing viral lethal mutagenesis, as illustrated in Figure 4*B*. Since HIV-1 already harbors powerful mutation synthesis and rapid evolution capability that enables the virus to escape from various anti-viral selective pressures such as host immune selection and antiviral agent treatments (58, 59, 60, 61), we may envision that even a modest increase in HIV-1 mutagenesis could readily drive HIV-1 toward error catastrophe. More specifically, as illustrated in Figure 4*B*, first, the incorporation of an RNA mutagen such as NHC-TP by cellular RNA polymerase II during viral transcription from the HIV-1 promoter encoded in the proviral DNAs integrated into host chromosomes could yield viral particles harboring viral RNA genomes containing NHC-MPs from the virus-producing cells treated with the RNA mutagen. Then, when these produced viral particles infect new target cells, HIV-1 RT can initiate C to T transition mutations during the first (−) strand DNA synthesis from the RNA genomes containing NHC-MPs. By repeating the mutagenic replication cycles, the C to U mutations can be accumulated in the viral RNA genomes, leading to the lethal mutagenesis. However, the antiviral effect of NHC against HIV-1 has not been reported, likely because the lethal mutagenesis phenotype requires long-term viral cultures in the presence of NHC. This lethal mutagenesis antiviral concept can be virologically tested against HIV-1 with future NHC-like RNA mutagens. Overall, given that the intrinsic hypermutability of HIV-1 may already approach its lethal mutagenesis threshold, this proof-of-concept mechanistic study using NHC-TP as a model compound suggests that RNA mutagens could serve as a viable platform for the development of therapeutics against HIV-1 and other viruses that use DdRps.

## Experimental procedures

### DNA templates

The dsDNA template used in T7 RNA polymerase mediated IVT reactions was originated form the HIV-1 pNL4-3 plasmid that encodes the entire NL4-3 HIV-1 genome in pUC plasmid backbone (62). A 328 nt region of the 5′ long terminal repeat part of pLN4-3 was amplified with addition of the T7 phage promoter at the beginning of the sequence with Phusion High-Fidelity DNA Polymerase (New England Biolabs, Cat. M0530 L) according to the manufacturer’s protocol. Primers used (Integrated DNA Technologies) are listed in Table S1 Section 1. The cycling conditions used were an initial denaturation at 98 °C for 30 s, 35 cycles: 98 °C for 10 s, 61 °C for 30 s, 72 °C for 30 s, and a final extension of 72 °C for 10 min. Their size is controlled by E-Gel EX Agarose Gels (Invitrogen, ThermoFisher Scientific, Cat. G401001). The dsDNA template for the HeLa cell nuclear extract-mediated IVT reactions was originated from pNL4-3. A 2346 nt region encoding the entire HIV-1 5′ LTR promoter was amplified from pLN4-3 with Phusion High-Fidelity DNA Polymerase (New England Biolabs, Cat. M0530 L) according to the manufacturer’s protocol and then purified by PCR purification (Qiagen, Cat. 28104) with the primers shown in Table S2 Section 1. The cycling conditions used were an initial denaturation at 98 °C for 30 s, 35 cycles: 98 °C for 10 s, 61 °C for 30 s, 72 °C for 30 s, and a final extension of 72 °C for 10 min. Their size is controlled by E-Gel EX Agarose Gels (Invitrogen, ThermoFisher Scientific, Cat. G401001).

### T7 polymerase-based IVT and product quantitation

IVT reactions were performed in three independent replicates for each condition, with T7 RNA polymerase (New England Biolabs, Cat. M0251S) with 4 μg of DNA template, for an incubation of 2 h at 37 °C, according to the manufacturer’s protocol under three different rNTP conditions. The first condition 3 rNTPs: 859 μM GTP, 2990 μM ATP and 460 μM UTP. The second condition four rNTPs: 66 μM CTP, 859 μM GTP, 2990uM ATP, 460 μM UTP. The third condition 3 rNTPs with NHC-TP: 859 μM GTP, 2990 μM ATP, 460 μM UTP and 102.8 μM NHC-triphosphate (1 mg, MedChemExpress). We previously reported these cellular rNTP concentrations were previously reported in Kennedy *et al*., 2010 (17) and for NHC-TP in Ehteshami *et al*., 2025 (18).

### DNase treatment and qRT PCR for T7 RNA polymerase mediated IVT reactions

The RNA products obtained from IVT reactions were extracted with RNeasy Mini Kit (Qiagen, Cat. 74104) according to the manufacturer’s protocol. Then, it is treated with TURBO DNA-free Kit (Invitrogen, ThermoFisher Scientific, Cat. AM1907), 100 μl (200 units) of DNAse for 250 μl reaction. Copy numbers of the DNA template in IVT reactions were quantified by qPCR with LightCycler 480 Probes Master (Roche), and the copy numbers of the RNA products in the IVT reactions were determined with the qScript XLT One-Step RT-qPCR (QuantaBio, Cat. 95132-500) on the LightCycler 480 Instrument (Roche) according to the manufacturer’s protocol. pNL4-3 was used for the copy number control with primers and probes shown in Table S1 Section 2. The cycling conditions for qPCR used were an initial denaturation at 95 °C for 5 min, 45 cycles: 95 °C for 10 s, 60 °C for 30 s, 72 °C for 1 s. The cycling conditions for RT-qPCR used were a reverse transcription at 50 °C for 10 min, a polymerase activation at 95 °C for 1 min and 45 cycles: 95 °C for 10 s, 60 °C for 60 s. The data have been put into graphical form with GraphPad Prism (version 10.5.0, GraphPad Software). RNA products were also examined using agarose gel electrophoresis. Agarose gel electrophoresis was performed by using a RiboRuler High Range RNA Ladder (ThermoFisher, Cat. SM1823) and a RNA Gel Loading Dye 2X (ThermoFisher, Cat. R0641) and revealed using ethidium bromide with ChemiDoc Touch Imaging System (Bio-Rad).

### Reverse transcription by HIV-1 RT and amplification, and product quantitation

Reverse transcription was performed with High-Capacity cDNA Reverse Transcription Kit (ThermoFisher Scientific, Cat. 437 4967) with 211.5 nM of HIV RT, 10 ng of RNA IVT products, and reverse primer (Table S1 Section 3). The produced cDNAs were amplified with Phusion High-Fidelity DNA Polymerase (New England Biolabs, Cat. M0530 L) according to the manufacturer’s protocol, and the amplified DNA products were purified by PCR purification (Qiagen, Cat. 28104). The level of DNA is quantified in three technical replicates by qPCR with LightCycler 480 Probes Master (Roche) on the LightCycler 480 Instrument (Roche) according to the manufacturer’s protocol. pNL4-3 was used as a copy number control with the primers used (Table S1 Section 4). The cycling conditions used were an initial denaturation at 95 °C for 5 min, 45 cycles: 95 °C for 10 s, 60 °C for 30 s, 72 °C for 1 s. The data have been put into graphical form with GraphPad Prism (version 10.5.0, GraphPad Software). Amplified DNA products were also examined using agarose gel electrophoresis. Agarose gel electrophoresis was performed by using a 1 Kb Plus DNA Ladder (Invitrogen, Cat. 10787018) and 6x DNA Loading Dye (ThermoFisher, Cat. R0611) and revealed using ethidium bromide with ChemiDoc Touch Imaging System (Bio-Rad).

### Library DNA preparation and illumina sequencing

Amplified DNA samples were purified by gel purification (Qiagen, Cat. 28704) after running 1% agarose gel. DNA libraries were prepared with Nextera XT DNA library Prep (Illumina, FC-131-1096) for 1 ng DNA input; tagmentation, amplifying and clean up library. Their quality was verified by Bioanalyser 2100 (Agilent) and their quantity for the concentration all amplicons were quantified using the Qubit dsDNA HS Assay Kit (Life Technologies) and Qubit four Fluorometer (ThermoFisher Scientific). After that, the DNA library was pooled and diluted at 8 PM of concentration for the sequencing. The Illumina sequencing was performed with Illumina Miseq version 2 with 2 × 151 paired-end sequencing.

### DNA product sequencing and data analysis

Sequencing data were analyzed with tools on the Galaxy server (usegalaxy.org) (63). First, we performed a quality control check on raw sequence data of the sequencing with the tool FastQC (https://www.bioinformatics.babraham.ac.uk/projects/fastqc/). Then, with trimmomatic tool (64), we cut adapter and other Illumina-specific sequences from the read and the paired reads were kept for the rest of the analysis. The paired reads are mapped with the reference genome (Fig. S1), the original short sequence of the vector (210 bp), with the tools BWA-MEM2 (65). The aligned sequence is used to identify mutation with the call on variant – Ivar variant method (20). This method mainly detects frequent and abundant mutations as in consensus sequence, present at higher frequencies (>3%). With that information, calculations were made to known and plot in Figure 3. The data have been put into graphical form with GraphPad Prism (version 10.5.0, GraphPad Software). The details of data are in Table S3. The overall mutation rates are calculated with: (total mutated reads)/(Total sequence reads) according to the paper Rawson *et al*. (66). For percentage of mutation for all C sites: (mutated reads at C sites/total reads at C sites). And for the mutation frequency at each specific C sites of the RNA template are calculated with: (mutated reads sequenced at specific site position)/(total reads at this specific site position).

### HeLa cell nuclear extract-based ITV

IVT is performed based on Dignam *et al*. paper (51), in three independent replicates, using HeLa cell nuclear extract prepared by the previously reported method (23). 20uL of reaction included 1.4 ug of DNA template, 10 ul of nuclear extract, 12 mM HEPES pH 7.9 at 25 °C, 12% (v/v) glycerol, 0.3 mM DTT, 0.12 mM EDTA, 60 mM KCI, 12 mM MgCl2. The physiological condition was tested with 66 μM CTP, 859 μM GTP, 2990 μM ATP, 460 μM UTP and/or 102.8 μM of NHC-triphosphate (MedChemExpress) as described above. The standard incubation was for 60 min at 30 °C. The RNA products were extracted with RNeasy Mini Kit (Qiagen) according to the manufacturer’s protocol. Then, they were treated with TURBO DNA-free Kit (Invitrogen, ThermoFisher Scientific, Cat. AM1907); 1 μl of DNAse in 50 μl reaction and incubated at 37 °C for 1 h according to the manufacturer’s protocol. Due to the cellular RNAs in the nuclear extract, the RNA concentration was measured by Nanodrop (ThermoFisher Scientific) to normalize the RNA contents for RT-qPCR. RNA copy numbers were determined in three technical replicates on the LightCycler 480 Instrument (Roche) with the qScript XLT One-Step RT-qPCR (QuantaBio, Cat. 95132-500) according to the manufacturer’s protocol. pNL4-3 was used as a copy number control and the primers used are in Table S2 Section 2. The cycling conditions used were a reverse transcription at 50 °C for 10 min, a polymerase activation at 95 °C for 1 min and 45 cycles: 95 °C for 10 s, 60 °C for 60 s. The data have been put into graphical form and performed a statistical test non-parametric with Kruskal-Wallis test and Dunn’s test with GraphPad Prism (version 10.5.0, GraphPad Software).

## Data availability

The workflow construct for the sequencing data analysis can be found at:https://usegalaxy.org/u/nathaliebiologie/w/workflow-mutagenesis

## Supporting information

This article contains supporting information.

## Conflict of interest

The authors declare that they have no conflicts of interest with the contents of this article.
