# Supplementary material for: DNA-dependent RNA polymerase incorporates β-D-N4-hydroxycytidine (NHC) linking Molnupiravir to host transcription-dependent mutagenesis
Source: J Biol Chem. 2026 Mar 26;302(5):111409. doi: 10.1016/j.jbc.2026.111409 (PMC13129383; doi:10.1016/j.jbc.2026.111409)

**Supporting Information**

**Legends of Supplementary Figures and Tables**

**Table S1: List of primers used during T7 polymerase-based study.** For each primer, their sequence and position on the pNL4-3 HIV-1 genome is specified. Also, the size of the sequence generated.

**Table S2: List of primers used during HeLa cell nuclear-based study.** For each primer, their sequence and position on the pNL4-3 HIV-1 genome is specified. Also, the size of the sequence generated.

**Figure S1: Nucleotide sequence of 210 long region in the DNA template analyzed by NGS in Figure 3.**

**Table S3: Sequencing data obtained with IVT products generated with 4 rNTP condition and 3 rNTP with NHC-TP condition by the Ivar variant method. (A)** For each condition, all data from the Ivar variant method are shown, related to mutation reads sequenced and their mutation rates. Mutation rates were calculated with (total mutation reads detected) / (total sequence reads) for each condition. Mutation rates data are presented in Mean ± Standard deviation. **(B)** Percentage of mutation at each of all C sites in the IVT products with 3rNTP with NHC-TP condition. These values were calculated: (mutated reads sequenced at each C site) / (total reads at the same C site), presented in Mean ± Standard deviation.

**Figure S2: qPCR analysis of T7 RNA polymerase-mediated IVT products before and after DNase treatment.** DNA template levels in RNA products determined by qPCR before and after DNase treatment. IVTs were conducted in triplicates and qPCR analysis was conducted in duplicates for each IVT product. Mean with SD are represented with bars.

**Figure S3: qPCR analysis of HeLa cell NE-mediated IVT products before DNase treatment.** DNA template levels in RNA products determined by qPCR before DNase treatment. IVTs were conducted in triplicates and qPCR analysis was conducted in duplicates for each IVT product. Mean with SD are represented with bars.

**Figure S4: NHC-TP and CTP concentration dependent IVT product.** The IVT reactions were conducted and analyzed as described in Figure ID except changing the concentration of the nucleotide substrates and the volume of IVT reaction (100uL after 1 hour) used. The concentration of the three other rNTP concentrations (ATP, GTP, and UTP) were fixed at 2.5mM, while NHC-TP and CTP concentrations were changed from 2.5mM, 1mM, 0.5mM, 0.25mM, and 0.1mM (lanes 1-5). The IVT products with CP were diluted by 1/50 while the ones with NHC-TP were loaded with no dilution for the product analysis in 1% agarose gel. L: RNA ladder.

**Table S1: List of primers used during T7 polymerase-based study.**

|  | **Name** | **Primers** | **Sequence of primers (5’-3’)** | **Size (bp)** | **Position (bp)** |
| --- | --- | --- | --- | --- | --- |
| **1** | DNA template | Forward | CCC **TAA TAC GAC TCA CTA TAG** GGT CTC TCT GGT TAG ACC | 328 | 454 |
|  |  | Reverse | CTC GCA CCC ATC TCT CTC C |  | 800 |
| **2** | RT-qPCR and qPCR for T7 polymerase-based ITV | Forward | GAG ATC TCT CGA CGC AGG AC | 100 | 678 |
|  |  | Reverse | CGC ACC CAT CTC TCT CCT TC |  | 778 |
|  |  | Probe | /56-FAM/ TTT GAC TAG /ZEN/ CGG AGG CTA /3IABkFQ/ |  | 761 |
| **3** | HIV RT for T7 polymerase-based ITV | Reverse | CTC GCA CCC ATC TCT CTC C | 328 | 800 |
| **4** | qPCR for T7 polymerase-based RT | Forward | GAG ATC CCT CAG ACC CTT TT | 199 | 601 |
|  |  | Reverse | CTC GCA CCC ATC TCT CTC C |  | 800 |
|  |  | Probe | /56-FAM/ TTT GAC TAG /ZEN/ CGG AGG CTA /3IABkFQ/ |  | 761 |

**Table S2 : List of primers used during HeLa cell nuclear-based study.**

|  | **Name** | **Primers** | **Sequence of primers (5’-3’)** | **Size (bp)** | **Position (bp)** |
| --- | --- | --- | --- | --- | --- |
| **1** | DNA template for HeLa cell nuclear extract | Forward | CAT TTG CCC CTG GAG GTT CTG | 2 346 | 13 698 |
|  |  | Reverse | ACA CAG TCC TTC CTT ATT CC |  | 1 219 |
| **2** | HeLa ITV for RT-qPCR | Forward | GAG ATC TCT CGA CGC AGG AC | 100 | 678 |
|  |  | Reverse | CGC ACC CAT CTC TCT CCT TC |  | 778 |
|  |  | Probe | /56-FAM/ TTT GAC TAG /ZEN/ CGG AGG CTA /3IABkFQ/ |  | 761 |

**Figure S1: Nucleotide sequence of 210 long region encoded in the DNA template used in Figure 3.**

**
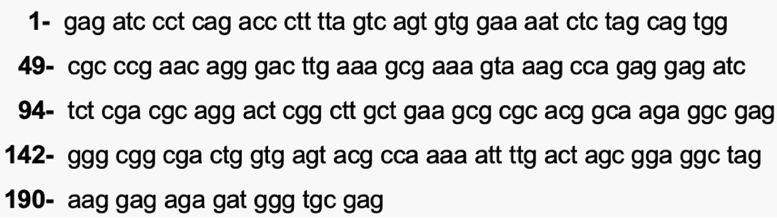
**

**Table S3: Sequencing data obtained with IVT products generated with 4 rNTP condition and 3 rNTP with NHC-TP condition by the Ivar variant method.**

**A.**

| **Condition** | **Total mutation reads** | | | | | | | | | **Total sequence reads** | | | **Mutation rate** |
| --- | --- | --- | --- | --- | --- | --- | --- | --- | --- | --- | --- | --- | --- |
|  | **C > T** | | | **C > G** | | | **T > G** | | | R1 | R2 | R3 |  |
|  | R1 | R2 | R3 | R1 | R2 | R3 | R1 | R2 | R3 |  |  |  |  |
| DNA template | 0 | 0 | 0 | 0 | 0 | 0 | 0 | 0 | 0 | 0 | 0 | 0 | 0 |
| 4 rNTP | 0 | 0 | 0 | 0 | 0 | 738 | 0 | 0 | 0 | 148052757 | 135596207 | 133426178 | 5.53x10^-6^ |
| 3 rNTP + NHC-TP | 18600887 | 15134517 | 15795967 | 0 | 0 | 0 | 0 | 0 | 137 | 175214695 | 144836469 | 148923338 | 1.06x10^-1^ ± 9.37x10^-4^ |

**B.**

| **Bases** | **Position** | **Mutated reads at specific position** | | | **Total reads at this position** | | | **Mutation frequency** |
| --- | --- | --- | --- | --- | --- | --- | --- | --- |
|  |  | R1 | R2 | R3 | R1 | R2 | R3 |  |
| C | 6 | 0 | 0 | 0 | 6936 | 1690 | 1182 | 0 |
| C | 7 | 0 | 0 | 0 | 25339 | 16714 | 15916 | 0 |
| C | 8 | 0 | 0 | 0 | 43070 | 31018 | 30145 | 0 |
| C | 10 | 0 | 0 | 0 | 44433 | 32024 | 31274 | 0 |
| C | 14 | 0 | 0 | 0 | 88163 | 74408 | 71178 | 0 |
| C | 15 | 0 | 0 | 0 | 95236 | 80044 | 76599 | 0 |
| C | 16 | 0 | 0 | 0 | 331331 | 291517 | 287488 | 0 |
| C > T | 24 | 200114 | 175075 | 173830 | 400886 | 348503 | 347352 | 5.01x10^-1^ ± 1.60x10^-3^ |
| C > T | 37 | 417641 | 46407 | 348552 | 794813 | 658163 | 661633 | 3.74x10^-1^ ± 2.63x10^-1^ |
| C > T | 39 | 415828 | 348455 | 348985 | 821386 | 681494 | 685332 | 5.09x10^-1^ ± 2.54x10^-3^ |
| C > T | 43 | 423628 | 351278 | 352672 | 856130 | 709251 | 714185 | 4.95x10^-1^ ± 7.51x10^-4^ |
| C > T | 49 | 473268 | 390849 | 394100 | 966594 | 800373 | 806937 | 4.89x10^-1^ ± 7.30x10^-4^ |
| C > T | 51 | 482152 | 398871 | 400402 | 1004083 | 828351 | 836930 | 4.80x10^-1^ ± 1.56x10^-3^ |
| C > T | 52 | 484337 | 400375 | 403442 | 1014351 | 836381 | 845306 | 4.78x10^-1^ ± 7.70x10^-4^ |
| C > T | 53 | 505823 | 419090 | 421730 | 1051667 | 868557 | 877256 | 4.81x10^-1^ ± 9.64x10^-4^ |
| C > T | 57 | 501362 | 415547 | 417689 | 1079978 | 893474 | 901762 | 4.64x10^-1^ ± 9.51x10^-4^ |
| C > T | 63 | 531194 | 437528 | 441839 | 1114188 | 914756 | 925039 | 4.78x10^-1^ ± 7.76x10^-4^ |
| C > T | 71 | 522644 | 428859 | 433637 | 1096447 | 897934 | 911576 | 4.77x10^-1^ ± 9.53x10^-4^ |
| C > T | 82 | 604370 | 501737 | 509076 | 1307686 | 1078438 | 1101166 | 4.63x10^-1^ ± 1.74x10^-3^ |
| C > T | 83 | 602992 | 501527 | 507098 | 1306794 | 1077606 | 1100378 | 4.63x10^-1^ ± 2.49x10^-3^ |
| C > T | 93 | 643494 | 530166 | 542292 | 1320568 | 1081710 | 1108868 | 4.89x10^-1^ ± 1.43x10^-3^ |
| C > T | 95 | 648348 | 534652 | 546585 | 1337970 | 1095780 | 1123836 | 4.86x10^-1^ ± 1.67x10^-3^ |
| C > T | 97 | 630810 | 520907 | 533645 | 1322915 | 1084225 | 1112512 | 4.79x10^-1^ ± 1.90x10^-3^ |
| C > T | 100 | 625990 | 517685 | 532789 | 1316927 | 1083531 | 1114816 | 4.77x10^-1^ ± 1.45x10^-3^ |
| C > T | 102 | 588293 | 487700 | 497729 | 1270741 | 1046277 | 1079517 | 4.63x10^-1^ ± 2.56x10^-3^ |
| C > T | 107 | 623232 | 517631 | 532515 | 1302177 | 1074225 | 1107832 | 4.80x10^-1^ ± 1.65x10^-3^ |
| C > T | 109 | 599868 | 500371 | 514094 | 1269778 | 1048760 | 1081425 | 4.75x10^-1^ ± 2.37x10^-3^ |
| C > T | 112 | 601659 | 502603 | 512663 | 1275461 | 1054924 | 1087623 | 4.73x10^-1^ ± 2.83x10^-3^ |
| C > T | 116 | 583575 | 488046 | 504073 | 1232423 | 1020370 | 1060428 | 4.76x10^-1^ ± 2.41x10^-3^ |
| C > T | 122 | 548240 | 456377 | 472291 | 1168153 | 967976 | 1006926 | 4.70x10^-1^ ± 1.33x10^-3^ |
| C > T | 124 | 550851 | 456922 | 474737 | 1165149 | 964491 | 1003918 | 4.73x10^-1^ ± 5.32x10^-4^ |
| C > T | 126 | 554612 | 460210 | 476997 | 1173109 | 971046 | 1010956 | 4.73x10^-1^ ± 1.05x10^-3^ |
| C > T | 128 | 557658 | 464326 | 484804 | 1180127 | 978086 | 1018731 | 4.74x10^-1^ ± 1.70x10^-3^ |
| C > T | 131 | 537513 | 449653 | 462498 | 1185071 | 980627 | 1021693 | 4.55x10^-1^ ± 3.16x10^-3^ |
| C > T | 138 | 507934 | 418018 | 430884 | 1101800 | 906772 | 946009 | 4.59x10^-1^ ± 3.19x10^-3^ |
| C > T | 145 | 480434 | 393300 | 407518 | 1049479 | 859496 | 898305 | 4.56x10^-1^ ± 2.33x10^-3^ |
| C > T | 148 | 477876 | 394157 | 403512 | 1058445 | 868588 | 906371 | 4.50x10^-1^ ± 4.45x10^-3^ |
| C > T | 151 | 487844 | 404988 | 420488 | 1042369 | 856059 | 893003 | 4.71x10^-1^ ± 2.54x10^-3^ |
| C > T | 161 | 422534 | 350404 | 364204 | 872338 | 720991 | 750503 | 4.85x10^-1^ ± 8.19x10^-4^ |
| C > T | 163 | 392406 | 326258 | 340945 | 827146 | 684702 | 712528 | 4.76x10^-1^ ± 2.05x10^-3^ |
| C > T | 164 | 395131 | 328621 | 340056 | 823021 | 680876 | 709165 | 4.81x10^-1^ ± 1.66x10^-3^ |
| C > T | 176 | 368777 | 306122 | 318650 | 723223 | 597366 | 624093 | 5.11x10^-1^ ± 1.32x10^-3^ |
| C > T | 180 | 332072 | 275827 | 288337 | 659855 | 547935 | 576733 | 5.02x10^-1^ ± 1.95x10^-3^ |
| C > T | 186 | 276383 | 233975 | 240609 | 599621 | 499197 | 525069 | 4.63x10^-1^ ± 5.43x10^-3^ |
| C | 207 | 0 | 0 | 0 | 4661 | 6294 | 5409 | 0 |

**Figure S2: qPCR analysis of T7 RNA polymerase mediated IVT products before and after DNase treatment.**

**
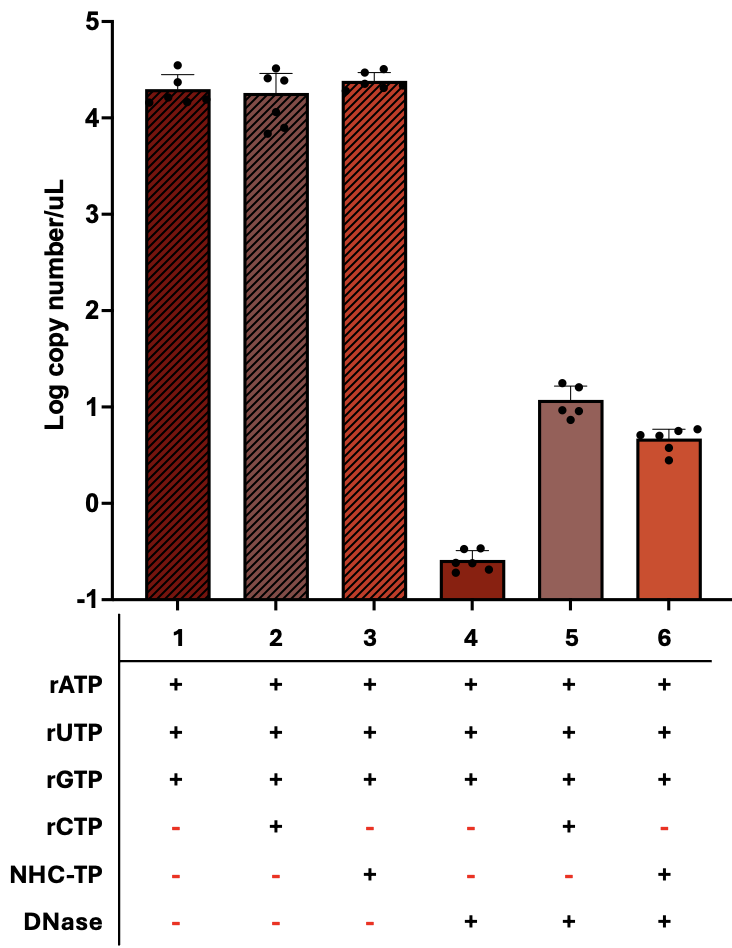
**

**Figure S3: qPCR analysis of HeLa cell nuclear extract mediated IVT products before DNase treatment.**

**
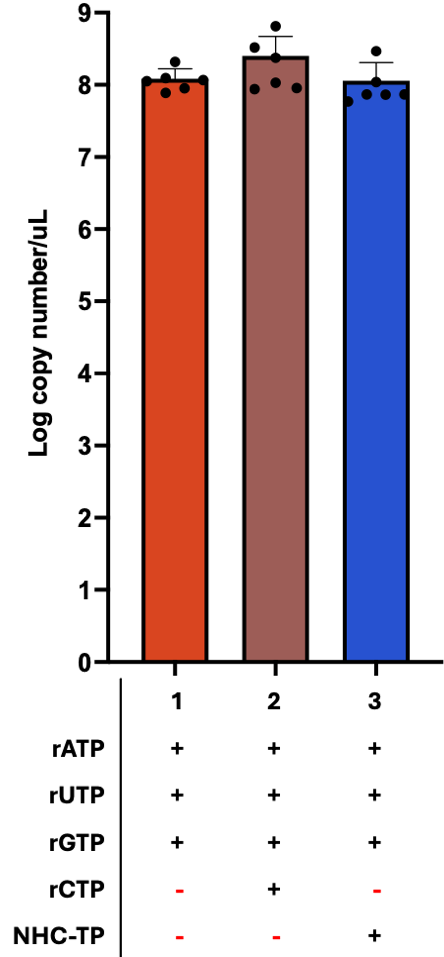
**

**Figure S4: NHC-TP and CTP concentration dependent IVT product.**


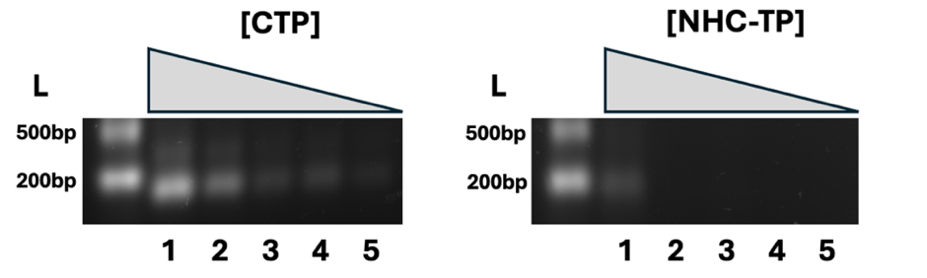

Supplement: Table S1 [file mmc1.doc]
